# Targeted Treatment of Metastatic Triple-Negative Breast Cancer: A Systematic Review

**DOI:** 10.1155/2024/9083055

**Published:** 2024-07-11

**Authors:** Anna Martha Hammershøi Madsen, Rikke Helene Løvendahl Eefsen, Dorte Nielsen, Iben Kümler

**Affiliations:** Department of Oncology 54 B1 Herlev Hospital University of Copenhagen, Herlev Ringvej 75, DK-2730, Copenhagen, Denmark

## Abstract

**Introduction:**

Triple-negative breast cancer (TNBC) is a subgroup of breast cancer characterized by the absence of estrogen and the human epidermal 2 receptor and also a lack of targeted therapy options. Chemotherapy has so far been the only approved treatment option, and patients with metastatic cancer have a dismal prognosis with a median overall survival (OS) of approximately 14 months. Identification of druggable targets for metastatic TNBC is therefore of special interest.

**Methods:**

A systematic search was performed, to review the existing evidence on targeted therapies in metastatic TNBC.

**Results:**

A total of 37 phase 2/3 studies were identified, evaluating 29 different targeted agents. In this review, results on progression free survival (PFS) and OS are presented.

**Conclusion:**

In most of the studies included, no improvement was observed for neither PFS nor OS; however, a few studies did show improvement with targeted agents and have led to new treatment options in subgroups of patients. The antibody drug conjugate, sacituzumab govitecan, demonstrated superior PFS and OS in comparison to chemotherapy. Immunotherapy with checkpoint inhibitors such as atezolizumab and pembrolizumab is now recommended as a first-line treatment option for patients with expression a PD-L1 positive tumor. Finally, the poly adenosine diphosphate-ribose polymerase (PARP) inhibitors talazoparib and olaparib are recommended, as first-line treatment options in patients with metastatic breast cancer and a germline BRCA mutation, but an immune checkpoint inhibitor should be considered for the subset of these patients who are PD-L1 positive.

## 1. Introduction

Triple-negative breast cancer (TNBC) is characterized by the lack of expression of the estrogen and progesterone receptor, as well as the human epidermal receptor 2 (HER2). Treatment options to TNBC are limited in comparison to other subtypes of breast cancer. Although the cancer disease is heterogeneous at the molecular level [1], most subgroups carry a dismal prognosis with a median overall survival (OS) of approximately 14 months [2–4]. Chemotherapy is used for the treatment for patients with metastatic TNBC (mTNBC); however, if the patients have a germline BRCA mutation or a programmed cell death ligand (PD-L1) expression of the cancer cells, additional therapy options are available. For patients with a germline BRCA mutation (gBRCAm), a poly adenosine diphosphate-ribose polymerase (PARP) inhibitor, such as olaparib or talazoparib, can be used as a first-line treatment option according to the ESMO guidelines [5] although treatment with an immune checkpoint inhibitor (ICI) should be considered for the subset of these patients who are PD-L1 positive [6]. Recommended ICIs are pembrolizumab in combination with paclitaxel, nab-paclitaxel, or carboplatin plus gemcitabine or atezolizumab plus nab-paclitaxel, according to the ESMO guidelines [5]. However, following results from the Impassion131 which failed to demonstrate a significant improvement of PFS and OS, the indication of atezolizumab combined with chemotherapy as a first-line option was withdrawn by FDA [7].

In later lines, the trop-2-directed antibody drug conjugate (ADC), sacituzumab govitecan, was demonstrated in the ACENT trial to significantly improve PFS and OS in comparison to standard chemotherapy [8]. Consequently, sacituzumab govitecan is now approved by FDA and EMA and recommended in the ESMO clinical guidelines as the preferred treatment option after anthracyclines and taxanes in patients with mTNBC [5, 9, 10]. About 40% of TNBC patients have a positive PD-L1 expression [11], but only 5% of breast cancer patients carry a germline BRCA mutation [12]. Thus, for the majority of patients, chemotherapy remains the only treatment option. Identification of new potential druggable targets is therefore of great interest. Several trials evaluating different targeted therapy options, as well as immunotherapy, have been conducted.

We performed a systematic search to review the clinical trials using targeted- and immunotherapies in the management of mTNBC.

## 2. Methods

A systematic search was performed independently by the authors (AH and IK) [13].

**Figure 1:**
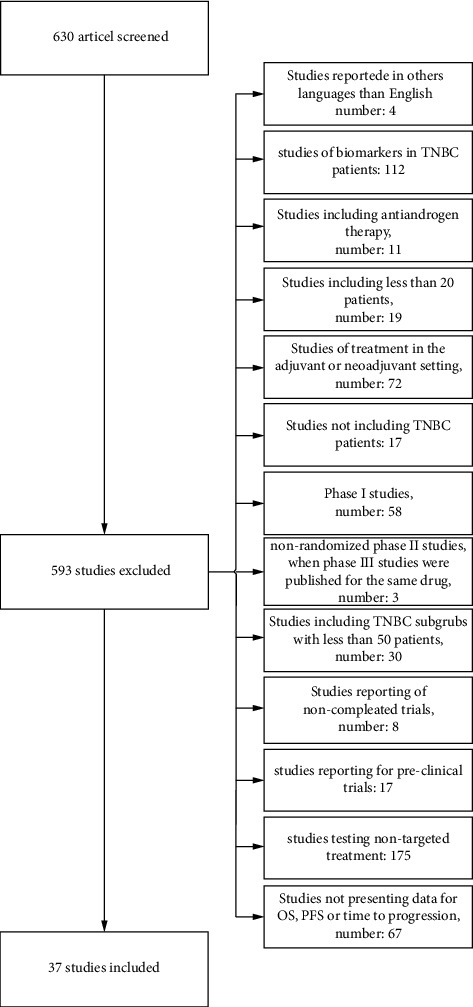
Flow diagram of the study selection.

The search terms “Triple negative,” “Triple Negative Breast Neoplasms,” “TNBC,” “Estrogen receptor negative,” “ER negative,” “Hormone receptor negative,” “HER2 negative,” “Human epidermal receptor 2,” “breast cancer,” “breast neoplasms,” and “breast carcinomas” were applied, and the “clinical trial” filter was activated. The data search was performed in the PubMed database and completed in April 2022. A total of 630 articles were identified, and all abstracts were examined. The screening process and reasons for exclusion of studies can be found in Figure 1.

## 3. Results

A total of 37 studies were included and listed in Table 1. Among included studies, 24 of them are randomized trials, 9 are phase III trials, and 15 are phase II trials. 13 studies were single-arm phase II studies. A total of 29 different therapeutic agents were identified and listed in Table 2.

There were 10 studies testing treatment in first-line [6, 11, 14–21], 7 studies testing treatment in second-line or more [8, 22–27] and 20 studies testing treatment in both first and later lines [28–47]. A total of 4 studies included TNBC patients as a subgroup [15, 17, 23, 24], and 12 studies included less than 50 patients [25–28, 36, 38, 41, 42, 44–47]. None of them found encouraging results for OS and PFS and will not be mentioned in this section but can be found in Table 1. For the remaining 26 randomized phase II and phase III studies, data on PFS and OS will be presented. The antineoplastic drugs included antibody drug-conjugates (ADC), monoclonal antibodies, PARP inhibitors, and tyrosine kinase inhibitors, also known as small molecules.

### 3.1. Antibody Drug-Conjugates

#### 3.1.1. Sacituzumab Govitecan

In a phase 1/2 study, a total of 108 patients were enrolled and treated with sacituzumab govitecan [48]. This study was followed by the ASCENT trial, a phase III trial comparing sacituzumab govitecan with chemotherapy [8]. In this open-label, randomized study (*n* = 468), patients who had received two or more previous lines of chemotherapy, including taxane, in the metastatic setting could be enrolled. Patients were assigned 1 : 1 to receive sacituzumab govitecan (*n* = 235) or single-agent chemotherapy of the physician's choice (eribulin, vinorelbine, capecitabine, or gemcitabine) (*n* = 233). PFS was 5.6 months for patients treated with sacituzumab govitecan and 1.7 months for patients who received chemotherapy (HR: 0.41, 95% CI: 0.32–0.52, *P* < 0.001) [8]. The median OS was 12.1 months in the group of patients receiving sacituzumab govitecan, while it was 6.7 months among patients treated with standard single-agent chemotherapy (HR: 0.48, 95% CI: 0.38–0.59, *P* < 0.001) [8].

### 3.2. Checkpoint Inhibitors

#### 3.2.1. Atezolizumab

The Impassion130 study was designed to evaluate the efficacy and safety of atezolizumab together with nab-paclitaxel as a first-line treatment [11, 49, 50]. This randomized, double blinded, placebo controlled, phase III trial included 902 patients, assigned in a 1 : 1 ratio to receive atezolizumab and nab-paclitaxel, or placebo and nab-paclitaxel. Patients were stratified according to PD-L1 expression on tumor-infiltrating immune cells. In the ITT population, PFS was 7.2 months with atezolizumab and 5.5 months with placebo (HR: 0.80, 95% CI: 0.69–0.92, *P*=0.002) [11]. Among the PD-L1-positive subgroup, PFS was 7.5 months with atezolizumab, while it was 5.0 months with placebo (HR: 0.62, 95% CI: 0.49–0.78, *P* < 0.001) [11]. In the ITT population, OS was 21.0 months among patients treated with atezolizumab and 18.7 months among patients treated with placebo (HR: 0.87, 95% CI: 0.75–1.02, *P*=0.077) [49, 50]. Exploratory analysis for the PD-L1-positive subgroup showed an OS of 25.4 months in the atezolizumab group and 17.9 months in the placebo group (HR: 0.67, 95% CI: 0.53–0.86) [50]. However, due to the prescribed statical testing hierarchy, OS in the PD-L1-positive subgroup could not be formally tested [50].

Impassion131, a randomized, double blinded, placebo-controlled phase III trial, tested the effects of paclitaxel with or without atezolizumab as a first-line treatment [14]. Patients were stratified according to PD-L1 expression on tumor-infiltrating immune cells, and PFS was tested hierarchically first in the PD-L1-positive subgroup and then in the ITT population. A total of 651 patients were included and randomized 2 : 1 to receive treatment with paclitaxel plus atezolizumab or placebo. For the PD-L1-positive subgroup, PFS was 6.0 months in the atezolizumab group and 5.7 months in the placebo group (HR: 0.82, 95% CI: 0.60–1.12, *P*=0.20). In ITT population, PFS was 5.7 months and 5.6 months, respectively [14]. OS for the PD-L1-positive subgroup was 22.1 months in the atezolizumab group and 28.3 months in the placebo group (HR: 1.11, 95% CI: 0.76–1.64). For the ITT population, OS was 19.2 months and 22.8 months, respectively (HR: 1.12, 95% CI: 0.88–1.43) [14].

#### 3.2.2. Pembrolizumab

Following a phase II study including 170 patients (KEYNOTE-086) [51, 52], a phase III study of pembrolizumab as first-line therapy was published (KEYNOTE-355). This randomized, double blinded trial investigated the ability of pembrolizumab to enhance the effect of chemotherapy in TNBC [6, 53]. The study randomly assigned 847 patients 2 : 1 to receive treatment with either chemotherapy and pembrolizumab (*n* = 566) or chemotherapy and placebo (*n* = 281). Chemotherapy regimens consisted of three different options: nab-paclitaxel, paclitaxel, or gemcitabine plus carboplatin. Patients were stratified according to PD-L1 status at the baseline (combined positive score, CPS [54]), PD-L1-positive tumors being of CPS ≥ 1 or CPS >/ = 10, and PL-L1 negative tumors being of CPS<1. Among patients with PD-L1 CPS >/ = 10, PFS for patients in the pembrolizumab group was 9.7 months, and for patients in the placebo group, it was 5.6 months (HR: 0.65, 95% CI: 0.49–0.86, *P*=0*·*0012). Among patients with PD-L1 CPS ≥ 1, PFS was 7.6 months and 5.6 months, respectively (HR: 0.74, 95% CI: 0.61–0.90, *P*=0.0014). In the group of patients with PD-L1 CPS <1, PFS was 6.3 months with pembrolizumab and PFS was 5.6 months with placebo (HR: 1.08, 95% CI: 0.77–1.53). In the ITT population, PFS was 7.5 months and 5.6 months, respectively (HR: 0.82, 95% CI: 0.69–0.97) [6]. OS in the group of patients with PD-L1 CPS ≥10 was 23.0 months with pembrolizumab and 16.1 months with placebo (HR: 0.73, 95% CI: 0.55–0.95, *P*=0.019). Among patients with PD-L1 CPS ≥1 OS was 17.6 months and 16.0 months, respectively (HR: 0.86, 95% CI: 0.72–1.04, *P*=0.11). No data of OS were reported for patients with PD-L1 CPS <1. For the ITT population, OS was 17.2 months with pembrolizumab and 15.5 months with placebo (HR: 0.89, 95% CI: 0.76–1.05) [53].

Following KEYNOTE-355, KEYNOTE-119 was conducted to compare pembrolizumab to chemotherapy in second- or third-line setting. This randomized, open-label, phase III trial included 622 patients previously treated with one or two lines of antineoplastic therapies, including anthracyclines or taxanes for metastatic disease [22]. Patients were randomly assigned 1 : 1 to receive pembrolizumab (*n* = 312) or chemotherapy by investigator's choice (*n* = 310). Randomization was based on PD-L1 tumor status. Only data of OS were reported. In the total population, OS was 9.9 months with pembrolizumab and 10.8 months with chemotherapy (HR: 0.97, 95% CI: 0.82–1.15). For the subgroup of patients with PD-L1 CPS ≥10, OS was 12.7 months with pembrolizumab and 11.6 months with chemotherapy (HR: 0.78, 95% CI: 0.57–1.06, *P*=0.057) [22]. OS for the subgroup of patients with PD-L1 CPS ≥1 was 10.7 months and 10.2, respectively (HR 0.86, 95% CI: 0.69–1.06, *P*=0.073) [22].

#### 3.2.3. Camrelizumab

In an open-label, randomized phase II trial, efficacy and safety of camrelizumab in combination with the tyrosine kinase inhibitor apatinib were evaluated in a cohort of 40 patients with metastatic or unresectable TNBC [28]. Patients eligible for the trial had received no more than two previous lines of systemic antineoplastic treatment for metastatic disease. Patients were allocated to treatment with camrelizumab with continued apatinib (*n* = 30) or intermittent apatinib (*n* = 10). PFS in the apatinib intermittent dosing cohort was 1.9 months (95% CI: 1.8–3.7), and in the apatinib continues dosing cohort, it was 3.7 months (95% CI: 2.0–6.4) [28]. OS was 9.5 months (95% CI: 2.7–14.8), and 8.1 months, respectively (95% CI: 4.0-not reached) [28].

### 3.3. Monoclonal Antibodies

#### 3.3.1. Bevacizumab

Bevacizumab has been evaluated in several smaller phase II trials in various combinations. Only phase III trials and randomized phase II trials are included here.

The RIBBON-1 trial was a placebo-controlled phase III study investigating the effect of adding bevacizumab to any standard first-line chemotherapeutic agent [15]. Prior to randomization, the investigator chose whether the patients were to receive capecitabine, an anthracycline-based or a taxane-based regimen. Totally 1.237 patients were included, and among these were 279 patients with TNBC. For TNBC patients treated with capecitabine, the addition of bevacizumab improved PFS from 4.2 months to 6.1 months (HR: 0.72, 95% CI: 0.49–1.06), and among TNBC patients treated with an anthracycline or a taxane, the addition of bevacizumab increased PFS from 6.2 months to 6.5 months (HR: 0.78, 95% CI: 0.53–1.15) [15]. For the entire study population, no statistically significant difference in OS was found [15].

The placebo-controlled phase II RIBBON-2 trial investigated the effect of adding bevacizumab to chemotherapy in second-line therapy [23]. Treating physicians were allowed to select any chemotherapy from a prespecified list, and subsequently, patients were randomized 2 : 1 to receive the chosen drug with placebo or bevacizumab. A total of 159 patients with TNBC were included in the exploratory subgroup analysis. PFS was 6.0 months with bevacizumab and 2.7 with placebo (*P*=0.0006) [23]. OS was 17.9 months and 12.6 months, respectively (*P*=0.0534) [23].

Another randomized trial (TANIA) investigated bevacizumab in second-line setting. As a part of this large, open-label, phase III trial, a subgroup of 106 TNBC patients who had progressed on first-line bevacizumab plus chemotherapy were randomly assigned to treatment with second-line chemotherapy in combination with bevacizumab or placebo [24, 55]. For TNBC patients in the bevacizumab group, PFS was 2.1, and for patients in the placebo group, PFS was 4.9 months (HR: 0.59, 95% CI: 0.40–0.88) [55]. No difference in OS was found [24].

#### 3.3.2. Onartuzumab

Onartuzumab was evaluated as first- or second-line treatment in a randomized, double-blinded, placebo-controlled phase II study [29]. Totally, 185 patients were included and randomly assigned to three different treatments: either onartuzumab combined with placebo and paclitaxel, onartuzumab in combination with bevacizumab and paclitaxel, or placebo together with bevacizumab and paclitaxel. Patients were allowed no more than one previous treatment in the metastatic setting. PFS among patients treated with onartuzumab combined with bevacizumab and paclitaxel was 5.4 months (95% CI: 3.75–5.55), among patients treated with the combination of onartuzumab, bevacizumab and paclitaxel, PFS was 7.3 (95% CI: 5.68–7.82) months, and in the group of patients that received placebo together with bevacizumab and paclitaxel, PFS was 7.2 months (95% CI: 5.52–9.26) [29]. OS was 13.4 months (95% CI: 9.76- not estimable), 14.7 months (95% CI: 10.58- not estimable), and 17.4 months (95% CI: 12.55- not estimable), respectively [29].

#### 3.3.3. Tigatuzumab

Tigatuzumab was investigated in combination with nab-paclitaxel as treatment in first and later lines in an open-label, randomized phase II trial (TBTRC 019) [30]. 64 patients were randomized 2 : 1 to receive nab-paclitaxel in combination with tigatuzumab or nab-paclitaxel as monotherapy. There was no limit of prior chemotherapy regimens, but patients must have received anthracyclines and taxanes in the neoadjuvant or adjuvant settings. PFS for patients treated with the combination of nab-paclitaxel and tigatuzumab was 2.8 months, and it was 3.7 months (95% CI: 2.3–5.7) [30] for patients receiving nab-paclitaxel monotherapy. Data of OS were not reported.

#### 3.3.4. Cetuximab

A large, randomized study including 173 patients investigated the effect of adding cetuximab to cisplatin in first- or second-line of treatment [31]. Patients were allowed to have received no more than one prior chemotherapy regimen for metastatic disease. Patients were randomized 2 : 1 to receive treatment with cisplatin plus cetuximab (maximum of six cycles) or cisplatin as monotherapy. On disease progression, patients treated with cisplatin alone were allowed to switch to cetuximab monotherapy or cetuximab plus cisplatin. A total of 31 patients switched to cetuximab containing therapy. PFS in the group of patients receiving cisplatin and cetuximab was 3.7 months, and in the group of patients receiving cisplatin as monotherapy, it was 1.5 months (*P*=0.032) [31]. OS for the cetuximab group was 12.9 months, and it was 9.4 months among patients treated with cisplatin alone (*P*=0.31) [31].

Cetuximab in combination with carboplatin was evaluated in a phase II, randomized, open-label trial including 102 patients previously treated with a maximum of three chemotherapy regimens, including adjuvant therapy (TBCRC 001) [32]. Patients were randomized to receive cetuximab initially alone, but with carboplatin added upon progression, or as concomitant therapy from the beginning. In the group of patients treated with cetuximab alone, TTP was 1.4 months (95% CI: 1.1–1.8), with carboplatin added at progression, TTP was 2.6 months (95% CI: 1.8–4.7), and in the concomitant cisplatin and carboplatin group, TTP was 2.1 months (CI 95%: 1.8–5.5) [32]. OS in patients treated with cetuximab and carboplatin added on progression was 7.5 months (95% CI: 5.0–11.6), and OS was 10.4 months (95% CI: 7.7–13.1) in the concomitant cisplatin and carboplatin group [32].

The combination of ixabepilone with or without cetuximab as the first-line treatment for metastatic disease was evaluated in an open-label, randomized phase II trial including 77 patients [16]. Forty patients were treated with ixabepilone alone, and 37 patients were treated with ixabepilone in combination with cetuximab. PFS was 4.1 months in both arms [16]. No data of OS were published.

### 3.4. PARP Inhibitors

#### 3.4.1. Iniparib

In 2011, a randomized phase II trial tested the efficacy of gemcitabine and carboplatin with or without iniparib in 123 TNBC patients treated with a maximum of two prior chemotherapy regimens for metastatic disease [34]. No data on BRCA status were provided. Patients received treatment with gemcitabine and carboplatin on days 1 and 8, with or without iniparib twice weekly on days 1, 4, 8, and 11, in three-week cycles. Treatment with iniparib resulted in PFS of 5.9 months, and for treatment without iniparib, PFS was 3.6 months (*P*=0.01) [34]. OS in the iniparib treatment group was 12.3 months, and without iniparib, OS was 7.7 months (*P*=0.01) [34]. These results led to the conductance of a randomized, open-label, phase III trial using the same study design, drugs, dosage, and treatment schedule as in the phase II study [35]. In phase III, 519 patients were included. Treatment with iniparib resulted in PFS of 5.1 months, and for treatment without iniparib, PFS was 4.1 months (*P*=0.027) [35]. OS in the iniparib treatment group was 11.8 months, and without iniparib, OS was 11.1 months (*P*=0.28) [35].

#### 3.4.2. Veliparib

Veliparib was investigated in a randomized phase II study, in which the addition of veliparib to cyclophosphamide was compared to cyclophosphamide alone [36]. A total of 45 patients were included. Prior therapy for metastatic disease was allowed, and median three prior chemotherapy regimens had been given. PFS in the group of patients treated with cyclophosphamide combined with veliparib was 2.1 months, and it was 1.9 months with cyclophosphamide alone (HR not reported) [36]. No data of OS were reported.

### 3.5. Small Molecules

#### 3.5.1. Sorafenib

Sorafenib was investigated as the first-line therapy in a randomized, double-blinded, phase II trial, comparing paclitaxel in combination with sorafenib versus paclitaxel with placebo [17]. Totally 237 patients were included, and among these were 94 patients with TNBC. In this subgroup, PFS was 5.6 months with sorafenib and 5.5 months with placebo (HR: 0.856, CI: 0.504 to 1.454) [17]. Data of OS were not reported.

#### 3.5.2. Sunitinib

In a randomized, open-label, phase II trial including 213 patients with TNBC, sunitinib was compared to investigator's choice of single agent chemotherapy [39]. PFS for patients treated with sunitinib was 2.0 months, and for patients treated with chemotherapy, it was 2.7 months (*P*=0.888). OS was 9.4 months and 10.5 months, respectively (*P*=0.839) [39].

#### 3.5.3. Reparixin

Reparixin combined with paclitaxel was evaluated as the first-line treatment in the randomized, placebo-controlled phase II trial (fRida) [18]. A total of 123 patients were randomized 1 : 1 to receive paclitaxel and reparixin (*n* = 62) or paclitaxel and placebo (*n* = 61). For the addition of reparixin to paclitaxel, PFS was 5.5 months and OS was 16 months. With placebo, PFS was 5.6 months (*P*=0.5996) and OS was 17.4 months (*P*=0.7059) [18].

#### 3.5.4. Trilaciclib

A multicenter, randomized, open-label phase II trial tested the neoplastic effect of adding trilaciclib to treatment with gemcitabine and carboplatin [43, 56]. In addition, the trial tested the ability of trilaciclib administrated before chemotherapy to protect the bone marrow from toxic effects of chemotherapy. The trial included 102 patients with locally recurrent or metastatic TNBC, who had received no more than two previous lines of chemotherapy. Patients were randomized 1 : 1 : 1 to three groups: group 1 received treatment with carboplatin and gemcitabine on days 1 and 8, patients in group 2 were treated with trilaciclib prior to carboplatin and gemcitabine on days 1 and 8, and group 3 received trilaciclib on days 1 and 8, and trilaciclib administrated before carboplatin and gemcitabine on days 2 and 9. The study found no significant differences regarding myelosuppression endpoints, when adding trilaciclib to treatment with gemcitabine and carboplatin in group 2 and group 3 [43]. PFS was 5.7 months for group 1, 9.4 months for group 2 (*P*=0.2099), 7.3 months for group 3 (*P*=0.1816), and for groups 2 and 3 combined, PFS was 9.0 months (*P*=0.1291). For group 1, OS was 12.6 months, OS was not reached in group 2 (*P*=0.0016), for group 3, OS was 17.8 months (*P*=0.0004), and for groups 2 and 3 combined, OS was 19.8 months (*P* < 0.0001) [56].

#### 3.5.5. Ipatasertib

The addition of ipatasertib to first-line paclitaxel was tested in the LOTUS trial. This double-blinded, randomized phase II trial included 124 patients, randomized 1 : 1 to receive paclitaxel plus placebo, or paclitaxel plus orally administrated ipatasertib [19, 57]. Randomization was stratified according to tumor PTEN status (these results are not presented here). In the ITT population, PFS was 6.2 months for patients treated with ipatasertib and 4.9 months for patients treated with placebo (*P*=0.037) [58]. OS in the ITT population was 25.8 months for patients treated with ipatasertib and 16.9 months for patients treated with placebo (HR: 0.80, 95% CI: 0.50–1.28) [57].

#### 3.5.6. Capivasertib

The safety and efficacy of capivasertib was evaluated in the PAKT trial [20]. This double-blinded, placebo-controlled, randomized phase II trial randomly assigned 140 patients with mTNBC 1 : 1 to receive the first-line treatment with paclitaxel and capivasertib or paclitaxel and placebo [20]. Results were presented for the ITT population and for a subgroup of patients with genetic alterations in tumor tissue of PIK3CA, PTEN, and AKT1 (the latter will not be presented here). In the ITT population, PFS was 5.9 months for patients treated with paclitaxel and capivasertib and 4.2 months with for patients treated with paclitaxel and placebo (*P*=0.06). OS in the ITT population was 19.1 with capivasertib and 12.6 months with placebo (*P*=0.04).

#### 3.5.7. Cobimetinib

In a phase II, randomized, three cohort trial (COLET), cobimetinib plus chemotherapy was evaluated with or without atezolizumab as the first-line treatment in 169 mTNBC patients [21]. The effect of cobimetinib was investigated in three cohorts; cohort I (*n* = 106): paclitaxel with cobimetinib, or paclitaxel with placebo, cohort II (*n* = 32): cobimetinib, atezolizumab, and paclitaxel, and cohort III (*n* 31): cobimetinib, atezolizumab, and nab-paclitaxel.

PFS for cohort I was 5.5 months for paclitaxel with cobimetinib and 3.8 months for paclitaxel with placebo (HR: 0.73, 95% CI: 0.43–1.24, *P*=0.25). PFS was 3.8 months in cohort II and 7.0 months in cohort III. OS was 16.0 months for paclitaxel with cobimetinib and 19.6 months for paclitaxel with placebo (HR: 1.05, 95% CI: 0.55–2.01). OS was 11 months in cohort II and not evaluable (NE) in cohort III [21].

## 4. Discussion

This systematic review of target treatment for mTNBC identified a total of 37 phase 2 and 3 studies, evaluating 29 different targeted agents. With this review, we demonstrate that several attempts have been made to improve the treatment options for patients with mTNBC, but the majority of these have been disappointing in regard with improved OS or PFS. Until very recently, no clinical trials have changed the landscape of mTNBC significantly; however, the emergence of antibody-drug conjugates (ADCs) is an exciting development. ADCs are antineoplastic drugs consisting of an antibody specific for a tumor antigen that is linked to a cytotoxic agent [59]. This structure allows ADCs to deliver their cytotoxic agent to cancer cells, without harming healthy cells [59]. Consequently, ADCs provide the basis for rapidly developing strategies to treat mTNBC. Presently, 19 ongoing trials are registered on the website https://www.clinicaltrials.gov, evaluating a total of 13 different ADCs for mTNBC [60].

This review presents results for the first phase III trial, the ACENT trial, of a promising ADC; sacituzumab govitecan. This ADC consists of an antibody against the human trophoblast cell-surface antigen 2 (trop-2) coupled to the topoisomerase I inhibitor SN-38 [8]. In the ACENT trial, sacituzumab govitecan demonstrated a significantly improved PFS and OS in comparison to standard chemotherapy [8] and was the first ADC to be approved by the FDA and EMA for treatment of mTNBC in second line or beyond [9, 10]. Sacituzumab govitecan is currently undergoing extensively clinical testing, and furthermore, it is the first ADC to be evaluated in the adjuvant setting in a large, randomized phase III trial (clinicaltrial.gov, identifier NCT04595565).

A subset of patients with TNBC expresses PD-L1, and they are candidates for anti-PD-L1/PD-1 therapies [11]. Atezolizumab is a PD-L1 inhibitor, and in combination with nab-paclitaxel, it is approved as the first-line treatment of mTNBC [5, 61]. The approval was based on the Impassion 130 study, as this phase III trial demonstrated that the combined treatment of atezolizumab and nab-paclitaxel improvement PFS by 2.5 months and OS by 7.5 months, compared to monotherapy with nab-paclitaxel [50]. However, following the approval data from Impassion130, the Impassion131 was published and raised question regarding the treatment efficacy. In Impassion131, patients were treated with paclitaxel instead of nab-paclitaxel, and the study did not demonstrate an improvement in either PFS or OS for the addition of atezolizumab in comparison to monotherapy with paclitaxel [14]. As a consequence, the FDA no longer recommends the use of atezolizumab as the first-line therapy in mTNBC [7]. Currently, the use of atezolizumab in combination with nab-paclitaxel is still recommended in the Ema and ESMO guidelines ^5,10^. .

Pembrolizumab, in combination with the first-line chemotherapy for patients with mTNBC and a PD-L1 positive tumor (CPS ≥ 10), has also achieved FDA and EMA approval and is recommended in the ESMO guidelines as the first-line treatment in mTNBC with CPS ≥ 10 [5, 62, 63]. However, the phase III trial, KEYNOTE-119, testing pembrolizumab versus chemotherapy in patients previously treated in the metastatic setting, failed to demonstrate a significant improvement of PFS and OS with pembrolizumab in comparison to placebo, neither in the total population, nor in the PD-L1 positive subgroup [22]. Therefore, pembrolizumab in later lines is not recommended in the ESMO guidelines [5].

Another monoclonal antibody that has undergone great clinical attention and been the subject of massive clinical research is the anti-VEGF antibody bevacizumab. Following accelerated FDA approval of bevacizumab in the metastatic setting in 2008 [64, 65], subsequent randomized studies have failed to prove any significant difference in OS when bevacizumab is added to various chemo regimens. In 2010, the FDA withdrew the metastatic breast cancer indication from bevacizumab [65, 66]. As for today, bevacizumab is not recommended in the ESMO guidelines [5].

In the subgroup of TNBC patients with BRCA1 and BRCA2 mutations, attention in recent years has been directed towards PARP inhibitors. Iniparib was the first PARP inhibitor to be investigated in breast cancer. Although a phase II study showed a statistically significant difference in OS, favoring the addition of iniparib to gemcitabine and carboplatin compared to placebo [34], no difference in OS was found when the larger phase III trial was published [35]. It has later been questioned if iniparib is in fact a true PARP inhibitor, which could partly explain the nonsignificant results of the phase III study [67, 68]. The PARP inhibitors olaparib and talazoparib were both evaluated in comparison to standard therapy in populations of patients with advanced breast cancer and a germline BRCA mutation. Both studies included a large subgroup of TNBC patients; however, none of them present any data for the TNBC subgroup. For that reason, these studies of olaparib and talazoparib are not included in this review [58, 59]. Based on these two trials, PARP inhibitors olaparib and talazoparib are currently recommended in the ESMO guidelines as the first-line treatment option for patients with germline BRCA positive metastatic breast cancer [5]. However, given the improved survival benefit with immunotherapy in KEYNOTE-355, ICI should be considered as the first-line option in patients who are PD-L1 positive [6].

Apart from the before mentioned studies, most of the trials included in this paper did not show significantly improved OS and PFS. However, a subset of therapeutic agents needs further evaluation. A very recent study of the cyclin-dependent kinase 4/6 (CDK4/6) inhibitor, trilaciclib, in combination with carboplatin and gemcitabine, showed numerically prolonged PFS and significant improvement of OS [43, 56]. Currently, a randomized phase III trial of trilaciclib versus placebo in patients receiving first- or second-line gemcitabine and carboplatin is registered at clinicaltrials.gov (identifier, NCT04799249). The LOTUS trial evaluated AKT-inhibitor, ipatasertib, in combination with paclitaxel as the first-line treatment in metastatic TNBC [19, 57]. The LOTUS trial found that treatment with ipatasertib significantly increased PFS and numerically increased OS. In the ITT population, compared to placebo [57], results of the LOTUS are consistent with results of the PAKT trial, that evaluate another AKT-inhibitor, capivasertib, in combination with paclitaxel as the first-line treatment. Capivasertib significantly increased OS and PFS in the ITT population compared to placebo [20]. Currently, both ipatasertib and capivasertib are registered in clinical phase III trials (clinicaltrials.gov, identifier NCT04177108 and NCT03997123, respectively).

## 5. Conclusion

As this review has elucidated, mTNBC is a subgroup of breast cancer with several failed attempts to improve treatment and prognosis. New treatment options have, however, been available in recent years, and new antineoplastic drugs are under rapid evaluation, hopefully improving treatment options, prognosis, and quality of life for mTNBC patients in the future.

## Figures and Tables

**Table 1:** Included studies.

Study	Treatment	*N*	Line of treatment	Study design	PFS	OS
*Antibody drug-conjugates*						
ASCENT trial, Bardia et al. 2021 [8]	**Sacituzumab govitecan** vs. chemotherapy of the physician's choice	468	Third-line	Open-label randomized phase 3 trial	**5.6 months** vs. 1.7 months (*P* < 0.001) HR not reported	**12.1 months** vs. 6.7 months (*P* < 0.001) HR not reported

*Monoclonal antibodies*						
IMpassion130, Schmid et al. 2021 [11, 49, 50]	**Atezolizumab** + nab-paclitaxel vs. placebo + nab-paclitaxel	902	First-line	Randomized, double blinded, placebo-controlled phase III trial	ITT population: **7.2 months** vs. 5.5 months (*P*=0.002) HR not reported PD-L1 positive subgroup: **7.5 months** vs. 5.0 months (*P* < 0.001) HR not reported	ITT population: **21.0 months** vs. 18.7 months (*P*=0.077). HR not reported PD-L1 positive subgroup: **25.4 months** vs. 17.9 months (HR: 0.67, 95% CI: 0.53–0.86)
IMpassion 131, Miles et al. 2021 [14]	**Atezolizumab** + paclitaxel vs. placebo + paclitaxel	651	First-line	Randomized, double blinded, placebo- controlled phase III trial	PD-L1-positive subgroup: **6.0 months** vs. 5.7 months (*P*=0.20) HR not reported ITT population: **5.7 months** vs. 5.6 months HR not reported	PD-L1-positive subgroup: **22.1 months** vs. 28.3 months (HR: 1.11, 95% CI: 0.76–1.64) ITT population **19.2 months** vs. 22.8 months (HR:1.12, 95% CI: 0.88–1.43)
KEYNOTE-355, Cortes et al. 2020 [6, 53]	**Pembroli-zumab** + chemotherapy vs. placebo + chemotherapy	847	First-line	Randomized, placebo-controlled, double-blinded, phase III trial	PD-L1 CPS ≥ 10: **9.7 months** vs. 5.6 months (*P*=0.0012) HR not reported PD-L1 CPS ≥ 1: **7.6 months** vs. 5.6 months (*P*=0.0014) HR not reported PD-L1 CPS <1: **6.3 months** vs. **5.6 months** (HR: 1.08, 95% CI: 0.77–1.53) ITT population: **7.5 months** vs. 5.6 months (HR: 0.82, 95% CI: 0.69–0.97)	PD-L1 CPS ≥ 10: **23.0 months** vs. 16.1 months (*P*=0.019) HR not reported PD-L1 CPS ≥ 1: **17.6 months** vs. 16.0 months (*P*=0.11) HR not reported ITT population: **17.2 months** vs. 15.5 months (HR:0.89, 95% CI: 0.62–0.91)
KEYNOTE-119, Winer et al. 2021 [22]	Pembrolizumab vs. chemotherapy	622	Second- or third-line	Randomized, open-label phase III trial	Not reported	PD-L1 CPS >/ = 10: **12.7 months** vs. 11.6 months (*P*=0.057) HR not reported PD-L1 CPS >/ = 1: **10.7 months** vs. 10.2 months *P*=0.073 HR not reported ITT population: **9.9 months** vs. 10.8 months (HR: 0.97, 95% CI: 0.82–1.15)
Efficacy and safety of camrelizumab combined with apatinib, Liu et al. 2020 [28]	**Camrelizumab** + **apatinib** continuously vs. **camrelizumab** + **apatinib** intermittent dosing	40	First-, second-, or third- line	Open-label, randomized, phase II trial	**1.9 months** (95% CI: 1.8–3.7) vs. **3.7 months** (95% CI: 2.0–6.4) HR not reported	**9.5 months** (95% CI: 2.7–14.8), vs. **8.1 months** (95% CI: 4.0- NR) HR not reported
RIBBON-1, Robert et al. 2011 [15]	**Bevacizumab** **+** capecitabine vs. placebo + capecitabine -------------------- **bevacizumab** **+** anthracycline/taxane vs. placebo + anthracycline/taxane	279 (subgroup)	First-line	Randomized, double blind, placebo-controlled phase III trial	**4.2 months** vs. 6.1 months (HR: 0.72, 95% CI: 0.49–1.06) ---------------------- **6.2 months** vs. 6.5 months (HR: 0.78, 95% CI: 0.53–1.15)	Not reported
RIBBON-2, Brufsky et al. 2012 [23]	**Bevacizumab** **+** chemotherapy of the physician's choice vs. placebo + chemotherapy of the physician's choice	159 (subgroup)	Second-line	Randomized, placebo-controlled phase III trial	**6.0 months** vs. 2.7 months (*P*=0.0006) HR not reported	**17.9 months** vs. 12.6 months (*P*=0.0534) HR not reported
TANIA, Minckwitz et al. 2014 [24, 55]	**Bevacizumab** + chemotherapy vs. chemotherapy of the physician's choice	106 (subgroup)	Second-line	Open-label, randomized phase III trial	**4.9 months** vs. 2.1 months (HR: 0.59, 95% CI: 0.40–0.88)	Not reported
Onartuzumab and/or bevacizumab in combination with weekly paclitaxel, Diéras et al. 2015 [29]	**Onartuzumab** + placebo + paclitaxel, vs. **onartuzumab** + **bevacizumab** + paclitaxel, vs. placebo + **bevacizumab** + paclitaxel	185	First- or second-line	Randomized, double-blinded, placebo-controlled phase II study	**5.4 months** (95% CI: 3.75–5.55) vs. **7.3 months** (95% CI: 5.68–7.82) vs. **7.2 months** (95% CI: 5.52–9.26)	**13.4 months** (95% CI: 9.76-NE) vs. **14.7 months** (95% CI: 10.58- NE) vs. **17.4 months** (95% CI: 12.55-NE)
TBTRC 019, Forero-Torres 2015 [30]	**Tigatuzumab** + nab-paclitaxel vs. nab-paclitaxel	64	First- second- third- fourth- fifth- or sixth-line	Open-label, randomized phase II trial	**2.8 months** vs. 3.7 months (95% CI: 2.3–5.7)	Not reported
Cetuximab with cisplatin versus cisplatin alone, Baselga et al. 2013 [31]	**Cetuximab** **+** **cisplatin** vs. cisplatin	173	First- or second- line	Open-label, randomized phase II trial	**3.7 months** vs. 1.5 months (*P*=0.032) HR not reported	**12.9 months** vs. 9.4 months (*P*=0.31) HR not reported
TBCRC 001, Carey et al. 2012 [32]	**Cetuximab** with carboplatin added upon progression vs. concomitant **cetuximab** + carboplatin	102	First-, second-, third- or fourth- line	Open-label, randomized phase II trial	Time to progression: **2.6 months** (95% CI: 1.8–4.7) vs. **2.1 months** (CI 95%: 1.8–5.5)	**7.5 months** (95% CI: 5.0–11.6) vs. **10.4 months** (95% CI: 7.7–13.1)
Ixabepilone alone or with cetuximab, Trédan et al. 2015 [16]	**Ixabepilone** vs. **ixabepilone** + **cetuximab**	77	First-line	Open-label, randomized phase II trial	**4.1 months** vs. 4.1 months HR not reported	Not reported
Panitumumab, gemcitabine, and carboplatin, Yardley et al. 2016 [33]	**Panitumumab** + gemcitabine + carboplatin	71	First- or second-line	Single-arm phase II trial	**4.4 months** (95% CI: 3.2–5.5)	**11.6 months** (95% CI: 8.6–15.2)

*PARP inhibitors*						
Iniparib plus chemotherapy, O'Shaughnessy et al. 2011 [34]	**Iniparib** + gemcitabine + carboplatin vs. gemcitabine + carboplatin	123	First-, second- or third-line	Open-label, randomized phase II study	**5.9 months** vs. 3.6 months (*P*=0.01) HR not reported	**12.3 months** vs. 7.7 months (*P*=0.01) HR not reported
Iniparib plus chemotherapy, phase III O'Shaughnessy et al. 2014 [35]	**Iniparib** + gemcitabine + carboplatin vs. gemcitabine + carboplatin	519	First-, second- or third-line	Open-label, randomized phase III study	**5.1 months** vs. 4.1 months (*P*=0.027) HR not reported	**11.8 months** vs. 11.1 months (*P*=0.28) HR not reported
Cyclo-phosphamide and veliparib, Kummer et al. 2016 [36]	**Veliparib** + cyclo-phosphamide vs. cyclo-phosphamide	45	No limit of prior lines	Open-label, randomized phase II study	**2.1 months** vs. 1.9 months HR not reported	Not reported
Olaparib for metastatic breast cancer in patients with germline BRCA mutation, Robson et al. 2017 [37]	**Olaparib** vs. chemotherapy of the physician's choice	150	First-, second- or third-line	Randomized, open-label phase III trial	ITT population: **7.0 months** vs. 4.2 months, *P* ≤ 0.001 HR not reported No data available for the TNBC subgroup	Not reported

*Small molecules*						
Sorafenib in combination with paclitaxel, Gradishar et al. 2012 [17]	**Sorafenib** + paclitaxel vs. placebo + paclitaxel	94 (subgroup)	First-line	Randomized, double-blinded phase IIb trial	**5.6 months** vs. 5.5 months (HR: 0.86, CI: 0.50–1.45)	Not reported
Efficacy and safety of afatinib, Schuler et al. 2012 [38]	**Afatinib**	29	First- second- or third-line	Open-label, single-arm phase II study	**7.4 weeks** (95% CI: 5.6–10.1)	Not reported
Phase II trial of ENMD-2076, Diamond et al. 2012 [25]	**ENMD-2076**	41	Second-, third- or fourth- line	Single-arm, phase II clinical trial	**1.8 months** (95% CI: 1.8–3.9)	Not reported
Sunitinib versus standard of care, Curigliano et al. 2013 [39]	**Sunitinib** vs. chemotherapy of the physician's choice	213	First-, second- or third-line	Randomized, open-label, phase II trial	**2.0 months** vs. 2.7 months (*P*=0.888) HR not reported	**9.4 months** vs. 10.5 months (*P*=0.839) HR not reported
Phase II study of apatinib, Hu et al. 2014 [40]	**Apatinib**	56	First-, second- or third-line	Single-arm phase II study	**3.3 months** (95% CI: 1.7–5.0)	**10.6 months** (95% CI: 5.6–15.7)
Cabozantinib in TNBC, Tolaney et al. 2016 [41]	**Cabozantinib**	35	First-, second-, third or fourth-line	Single-arm phase II study	**2.0 months** (95% CI: 1.3–3.3)	Not reported
Adavosertib combined with cisplatin, Keenan et al. 2021 [42]	**Adavosertib** + cisplatin	34	First- or second- line	Single arm, phase II study	**4.9 months** (95% CI: 2.3–5.7)	**12.5 months** (95% CI: 7.5–14.9)
fRida, Goldstein et al. 2021 [18]	**Reparixin** + paclitaxel vs. placebo + paclitaxel	123	First-line	Randomized, placebo-controlled, phase II study	**5.5 months** vs. 5.6 months (*P*=0.5996) HR not reported	**16.0 months** vs. 17.4 months (*P*=0.7059) HR not reported
UCN-01 in combination with irinotecan, ma et al. 2013 [26]	**UCN-01** + irinotecan	22	Second-, third- or fourth-line	Single-arm phase II study	Time to progression: **1.7 months** (95% CI: 1.2–2.6)	**1.7 months** (95% CI: 3.8–19.0)
Trilaciclib plus chemotherapy versus chemotherapy alone, Tan et al. 2022 [43, 56]	Group 1: gemcitabine + carboplatin on days 1 and 8. Group 2: **trilaciclib** prior to gemcitabine on days 1 and 8 group 3: **trilaciclib** on days 1 and 8, + **trilaciclib** before gemcitabine + carboplatin on days 2 and 9	102	First-, second- or third-line	Open-label, randomized phase II trial	Group 1: 5.7 months group 2: **9.4 months** (*P*=0.2099) group 3: **7.3 months** (*P*=0.1816) groups 2 and 3 : **9.0 months** (*P*=0.1291) HR not reported	Group 1: 12.6 months group 2: **NR** (*P*=0.0016) group 3: **17.8 months** (*P*=0.0004) groups 2 and 3 : **19.8 months** (*P* < 0.0001) HR not reported
Everolimus and carboplatin combination, Singh et al. 2014 [44]	**Everolimus** + carboplatin	25	First-, second-, third- or fourth- line	Single-arm phase II trial	**3 months** (95% CI: 1.6–4.6)	**16.6 months** (95% CI: 7.3-NR)
LOTUS trial, Kim et al. 2021 [19, 57]	**Ipatasertib** + paclitaxel vs. placebo + paclitaxel	124	First-line	Double-blinded, randomized, phase II trial	**6.2 months** vs. 4.9 months (*P*=0.037) HR not reported	**25.8 months**, vs. 16.9 months (HR: 0.80, 95% CI: 0.50–1.28)
The PAKT trial, Schmid et al. 2019 [20]	**Capiversatib** + paclitaxel vs. placebo + paclitaxel	140	First-line	Double-blinded, randomized phase 2 trial	5.9 **months**, vs. 4.2 months *P*=0.06	**19.1 months** vs. 12.6 months. *P*=0.04
BKM120, Garrido-Castro et al. 2020 [45]	**Buparlisib**	50	First 30 patients: no limit of prior lines Last 20 patients: First-second- or third-line	Single arm, phase II study	**1.8 months** (95% CI: 1.6–2.3)	**11.2 months** (95% CI: 6.2–25)
Foretinib in patients with TNBC, Rayson et al. 2016 [46]	**Foretinib**	37	First- or second- line	Single-arm, phase II study	**1.9 months** (95% CI: 1.8–3.2)	Not reported
Tivantinib in patients with metastatic TNBC, Toleany et al. 2015 [27]	**Tivantinib**	22	Second-, third- or fourth-line	Single-arm, phase II study	**1.2 months** (95% CI: 1.0–1.4)	Not reported
COLET, Brufskey et al. 2021 [21]	Cohort I: **Cobimetinib** + paclitaxel vs. placebo + paclitaxel, cohort II: **cobimetinib** + atezolizumab + paclitaxel, cohort III: **Cobimetinib** + atezolizumab + nab-paclitaxel	169	First-line	Randomized phase II trial	Cohort I: **5.5 months** vs. 3.3 months (HR: 0.73, 95% CI: 0.43–1.24) cohort II: **3.8 months** (95% CI: 3.02–7.36) cohort III: **7.0 months** (95% CI: 3.65-12.81)	Cohort I: **16.0 months** vs. 19.6 months (HR: 1.05, 95% CI: 0.55–2.01) cohort II: **11.0 months** (95% CI 9.53-NE) cohort III: **NE**

*Others*						
Efficacy and safety of continuous infusion of RH-endostatin, Tan et al. 2021 [47]	**RH-endostatin** + platinum + gemcitabine, docetaxel, or pemetrexed	21	First-, second- or third-line	Single-arm, single-center, open-label trial	**8.8 months** 95% CI: 7.2–10.4)	**13.3 months** (95% CI: 11.6–15.0)

Abbreviations: OS: overall survival, PFS: progression-free survival, NR: not reached, NE: not estimated, HR: hazard ratio, *P*: *P* value, ITT: intention to treat, CI: confidence interval. *P* values or 95% CI interval is provided in the table, as well as in the text, in all the cases that these were available in the article.

**Table 2:** Therapeutic agents for targeted treatment in metastatic TNBC.

*Antibody drug-conjugates*	
Sacituzumab govitecan	Trop-2-directed antibody
*Monoclonal antibodies*	
Atezolizumab	Anti-PD-L1 antibody
Pembrolizumab	Anti-PD1 antibody
Camrelizumab	Anti-PD1 antibody
Bevacizumab	Anti-VEGF antibody
Onartuzumab	Anti-MET antibody
Tigatuzumab	Anti-DR5 antibody
Cetuximab	Anti-EGFR antibody
Panitumumab	Anti-EGFR antibody

*PARP inhibitors*	
Iniparib	PARP inhibitor
Veliparib	PARP inhibitor

*Small molecules*	
Sorafenib	Multikinase inhibitor targeting angiogenesis
Afatinib	EGFR inhibitor
ENMD-2076	Inhibitor of angiogenetic and mitotic kinases
Sunitinib	Inhibitor of receptor tyrosine kinases, including VEGFR, PDGFR, KIT, and CSF-1R23
Apatinib	Selective tyrosine kinase inhibitor targeting VEGFR2
Cabozantinib	Inhibitor of multiple tyrosine kinases, including MET and vascular endothelial growth factor receptor 2 (VEGFR2)
Adavosertib	Selective inhibitor of WEE1
Reparixin	Inhibitor of CXCR1
UCN-01	Check-point kinase 1 inhibitor
Trilaciclib	Inhibitor of cyclin-dependent kinase-4/6 (CDK4/6)
Everolimus	Selective inhibitor of mammalian target of Rapamycin (mTOR)
Ipatasertib	Selective AKT inhibitor
Capivasertib	Kinase inhibitor of all three AKT isoforms (AKT1, AKT2, AKT3)
Buparlisib	Pan-class I PI3K inhibitor
Foretinib	Multikinase inhibitor, primarily targeting MET
Tivantinib	Selective MET inhibitor
Cobimetinib	Selective MEK1/2 inhibitor

*Others*	
Recombinant human endostatin (RH-endostatin)	Inhibits angiogenesis

## Data Availability

No new data were created or analyzed in this study. Data sharing is not applicable to this article.
